# The Seven Ages of Man

**Published:** 1888-10-27

**Authors:** 


### The Doctor's Pulpit.

### THE SEVEN AGES OF MAN.

### IV.—The Soldier.

"Then a soldier, full of strange oaths . . . . jealous in honour . . . . seeking the bubble reputation."

To play the part of "flush" and virile manhood Shakespeare chooses the soldier. Why not the politician, the merchant, the lawyer, or the priest? Probably his choice was determined rather by dramatic than by philosophic considerations. The soldier makes the bravest show of all his contemporaries. His robust and handsome form, helped, where nature fails, by the cunning of the tailor's art; his brilliant clothing and accoutrements; his martial bearing and "big manly voice," accustomed to tones of command; his supreme consciousness of his own pre-eminent dignity—these all unite to constitute him a figure more full of dramatic possibilities than can be furnished by any other profession.

But whilst the choice of typical manhood has been determined by outward and visible circumstances, the true characteristics and animating motives common to the soldier and all other men are still given their due prominence. The warrior more than other men may be "full of strange oaths" and "bearded like the pard." He may be more than the lawyer, the doctor, or the priest, "jealous in honour" and "sudden and quick in quarrel," but when he seeks as his one great aim in life the "bubble reputation," and seeks it "even in the cannon's mouth," he shows at once his kinship with all mankind. His desire to be great and famous is the one peculiar "touch of nature" which makes him every man's "kin." That brings us to this point, that Shakespeare did not mean to affirm that one of the normal seven ages of man was a period of soldiership, but that at one stage of every man's life the all-compelling motive of action was love of fame, reputation. That stage of life, that natural human passion receives fit and dramatic illustration in the soldier, who is so resolved upon fame and proud distinction, that he will seek it "even in the cannon's mouth."

The hunger for public recognition is a characteristic of fully-developed manhood. The very young man is concerned with other things—love-making, pleasure, and the like. The man who has passed the sober age of fifty, unless he be well advanced on fame's highway, has usually given up the race, and contents himself with the duties and rewards of commonplace life. But the man of thirty or forty, who is conscious of decided powers and high courage, lifts his head high, and says to his own secret heart, "I also am a man, and my fellow-men shall sooner or later see and know what I can do." This period of fully-developed manhood is perhaps the most critical in the whole life. The reign of the passions is practically over. Reason has begun to assert its dominance. A choice has to be made of the way of life. Every intelligent man by this time sees that there are two chief planes on which life may be lived. In indicating these let no unphilosophic person imagine that the doctor is invading the region of the clergyman. The doctor is, indeed, speaking purely as a man, educated in medicine and science, who has used the spectacles furnished by such education to observe, as a man, all the ways of human life. There are, then, two leading planes on which life may be lived. The one is a plane which recognises the duty of living under and by the guidance of an authority external to man. The other is a plane which, avowedly or otherwise, recognises no such duty, but simply lives in that way which best pleases the natural impulses and desires. What has he whose purpose is fame and reputation to say on this point? Which of the two planes is he choosing? He is undoubtedly choosing the latter if his object be fame and reputation solely.

The divine has something to say to this; but the doctor, from a purely medico-scientific point of view, has also something to say. The clergyman recommends a certain way of life because he wishes to make his parishioner *virtuous* and happy; the doctor recommends a certain way of life because he desires to see his patient *healthy* and happy. It will be worthy of more than a passing consideration if the preacher of religion and the preacher of the gospel of bodily sanitation should agree to recommend the same plane of life. We will look at the lower plane first—that of living in accordance with the strongest natural impulses and desires. These strongest impulses may be neither coarse nor mean; on the contrary, they may be in a measure high and honourable. They may be such as to cause a man to seek first and chiefly a great name and a proud fame. The clergyman will say that such an object in life is not the highest. It may be high as compared with low objects, but it is incomparably low as compared with the highest. He will say also that fame, however great and splendid, can never satisfy the mind. But the doctor, studying man in his entirety, as physical, mental, and moral, also concludes that the living of life on the plane of self-pleasing is a complete failure as regards the most perfect bodily health, mental and moral development, and rational pleasure. If the intelligent study of physiology and the practise of the medical art declare anything, they declare this—that the normal and right condition for a man is not that of self-pleasing and self-enjoyment. Self-pleasing and self-seeking never lead to the highest perfection of physical, mental, and moral health. With regard to the second point of the clergyman, that these things do not satisfy the mind, physiology shows that the more a man indulges his merely natural desires, whatever they be, the more imperious do their demands become. Instead of their indulgence yielding a reasonable satisfaction, it excites a craving hunger, which makes satisfaction impossible. The most unhealthy body and mind a man can possess is one full of imperious cravings which can no longer be governed. That is a condition in which physical and mental insanity have reached their maximum.

The other of the two planes of life is that in which a man recognises that he owes one great duty to an intelligent Power external to himself. That duty is no less than the living of his whole life according to the directions of the Power, and for the rewards which the Power will bestow. The man yields voluntarily to an external authority, and resolves to be pleased with the wages of the service. The clergyman advises life on this plane on moral grounds, on what he calls eternal considerations. But physiology also advises it on grounds of health. This science recognises that every man, even the greatest, the clearest in intellect, the wisest in judgment, the strongest in will, is still what philosophers call a derived creature, a finite creature, limited, conditioned; not absolute, not independent—made, in fact, to depend on, to be guided by, to be subject to an absolute and final, as opposed to a conditioned and temporary intelligence. Religion and physiological science agree in this, that man, as man, is made to voluntarily choose and steadfastly do the will of God, and to take with pleasure the position, duties, and rewards in the world which naturally flow from such doing. Only by living life on this plane are the highest bodily and mental health and the fullest bodily and mental pleasure attainable.

Here then, finally, we are brought into deadly collision with Shakespeare's soldier and typical man, who clearly is not only one who yields to every impulse of the moment, swearing strange oaths, quarrelling on small provocation, yielding to momentary and silly jealousies, but who also deliberately chooses to live on the lower of the two chief planes of life. Of course all soldiers are not of this peculiar type. If we should seek the whole world over it would be impossible to find a man who more completely illustrated the life lived on the higher plane than a soldier who has recently died. That soldier was General Gordon. Many such soldiers could be named, though none more famous than he. In himself he constantly showed how all kinds of apparent opposites and extremes are made one by a single reconciling principle, the principle of devoting the whole life to the highest service of which man is capable. Shakespeare's swaggering and somewhat vulgar soldier, though taken as the type of robust manhood by so distinguished a student of human nature, can in no sense be held up as a model, but only as an example to be avoided. Gordon, because of his mastery of and submission to one single ruling principle of the highest nobility, may for ever serve as a burning and shining light to all generations.

